# High-definition optical coherence tomography imaging of melanocytic lesions: a pilot study

**DOI:** 10.1007/s00403-013-1387-9

**Published:** 2013-07-06

**Authors:** Marc A. L. M. Boone, Sarah Norrenberg, Gregor B. E. Jemec, V. Del Marmol

**Affiliations:** 1 Department of Dermatology, Hôpital Erasme, Université Libre de Bruxelles, 808 Route de Lennik, 1070 Bruxelles, Belgium; 2 Department of Dermatology, Health Sciences Faculty, Roskilde Hospital, University of Copenhagen, Universitetsvej 1, P.O. Box 260, 4000 Roskilde, Denmark

**Keywords:** Melanocytic skin lesion, Dysplastic nevi, Melanoma, Histopathology, Dermoscopy, Reflectance confocal microscopy, High-definition optical coherence tomography

## Abstract

High-definition optical coherence tomography (HD-OCT) is a non-invasive in vivo imaging technique with cellular resolution based on the principle of conventional optical coherence tomography. The objective of this study was to evaluate HD-OCT for its ability to identify architectural patterns and cytologic features of melanocytic lesions. All lesions were examined by one observer clinically and using dermoscopy. Cross-sectional HD-OCT images were compared with histopathology. En face HD-OCT images were compared with reflectance confocal microscopy (RCM). Twenty-six melanocytic lesions of 26 patients were imaged. Identification of architectural patterns in cross-sectional mode and cytologic features of pigmented cells in the epidermis, dermo-epidermal junction, papillary dermis, and superficial reticular dermis in the en face mode was possible by HD-OCT. HD-OCT provides morphological imaging with sufficient resolution and penetration depth to discriminate architectural patterns and cytologic features of pigmented cells in epidermis and dermis. The method appears to offer the possibility of additional three-dimensional structural information complementary to that of RCM, albeit at a slightly lower lateral resolution. The diagnostic potential of HD-OCT regarding malignant melanoma is not high enough for ruling out a diagnosis of malignant melanoma.

## Introduction

One of the challenging problems in clinical dermatology is the early detection of malignant melanoma. Clinical diagnosis may be difficult, and although dermoscopy is routinely used by many, melanoma may even lack dermoscopic hallmarks in some patients [11, 15, 30]. New methods for diagnosis of melanoma are therefore continuously being developed [1].

Most prominent among these is reflectance confocal microscopy (RCM) [9, 16, 20, 23, 25, 27]. Several studies have shown a positive correlation between confocal features and dermoscopy and histopathology [17, 20, 21, 23, 28]. As an adjuvant tool to dermoscopy, RCM improves the diagnostic accuracy and the differentiation between benign and malignant melanocytic lesions [13, 23, 29]. Like dermoscopy, RCM enables the non-invasive imaging of the skin in horizontal plane, but does this at a quasi-histologic resolution. Superficial structures such as the epidermis and papillary dermis can be visualized with high contrast to a theoretical depth of 250 μm [26]. In spite of this limited penetration, a recently published retrospective study suggests that RCM can also be used to diagnose nodular lesions except in fully ulcerated lesions or when pronounced hyperkeratosis [19].

High-definition optical coherence tomography (HD-OCT) represents a recent in vivo non-invasive imaging technique with cellular resolution [5–8]. Through a lateral and axial resolution of <3 μm, this technique permits the morphologic analysis of microanatomical structures of the skin in 3D. In addition HD-OCT has an improved penetration depth, providing images of individual cells, in theory up to 570 μm in depth [8]. Pigment can be visualized in HD-OCT and imaging differences of constitutive pigmentation in normal skin according Fitzpatrick’s skin phototypes was described [7]. It is therefore speculated that HD-OCT may be a relevant method for the diagnosis of melanocytic lesions.

As a first step to study the possibilities and limitations of HD-OCT in the analysis of melanocytic lesions, we have therefore studied the architectural pattern and cytologic feature analysis of melanocytic lesions comparing the findings with histopathology, RCM, and dermoscopy.

## Materials and methods

### Subjects

Twenty-six patients with 26 pigmented lesions who contacted a dermatologist (MB) in order to have the lesion excised were studied. All patients provided informed consent for imaging their lesion by HD-OCT, RCM, and dermoscopy prior to excision.

### Images acquisition procedure

#### Before excision, lesion images were recorded in the following order

1. Digital dermoscopy (Molemax3^®^) (Derma Medical System GmbH, Vienna, Austria).
2. Reflectance Confocal Microsopy (VivaScope 3000^®^, Lucid-Tech Inc., Henrietta, NY, USA; Mavig GmbH Munich, Germany): lesions were scanned with this handheld VivoScope^®^. Vivastacks were performed.
3. High-definition optical coherence tomography (Skintell^®^, AgfaHealthcare, Mortsel, Belgium).

#### Instruments details

Digital dermoscopy imaging was done with the Molemax3^®^ using optical magnification 30×. Confocal microscopy was done with the VivaScope^®^VS3000 hand-held confocal scanning laser microscope using a near-infrared laser at 830 nm. The used zoom field of view is 0.5 × 0.5 mm. In theory, the tissue penetration depth goes up to 250 μm, and the total light power at the tissue is <22 mW. Technical aspects are described elsewhere [9, 13, 16, 17, 21–29]. HD-OCT was done using Skintell^®^. This is a technique based on the principle of conventional OCT using a two-dimensional, infrared-sensitive (1,000–1,700 nm) imaging array for light detection. The focal plane is continuously moved through the sample (focus tracking). Moreover, the system is capable of capturing a cross-sectional image and an en face image in real time, as well as of fast 3D acquisition. The field of view is 1.8 × 1.5 mm. In theory, the tissue penetration depth goes up to 570 μm, and the total light power at the tissue is <3.5 mW. Instrument and imaging procedures are described elsewhere [5–8].

### Histopathology

All lesions were then excised and underwent histologic examination for diagnostic confirmation. Excisions were fixed in formalin and embedded in paraffin. After routine processing, slides were stained with hematoxylin and eosin. After histopathologic analysis by two board-certified histopathologists, the lesions of the 26 patients were classified according histopathological criteria [4, 10, 12].

Architectural patterns and cytologic features were described. Skin architecture was evaluated for regularity of the rete ridges, thinning or thickening of the epidermis and flattening of the dermal papillae. Melanocytes were evaluated for morphologic characteristics.

Lesions with single melanocytes at the dermo-epidermal junction, elongated papillae, and lentiginous pattern were classified as junctional nevus as well as lesions with small junctional nests located at the tips of the ridges. Lesions with nests at the junction and in the papillary dermis were classified as compound nevi, superficial type. Lesions with nests at the junction, in the papillary and reticular dermis were determined as compound nevi, deep type. Compactly aggregated melanocytic nest in the dermis are considered as dermal nevi. A blue nevus is characterized by the presence of ill-defined median to deep dermal proliferation of elongated or dendritic melanocytes.

Lesions were considered to be dysplastic if the World Health Criteria for the diagnosis of dysplastic nevi were fulfilled [10, 12]. The major criteria are (1) basilar proliferation of atypical melanocytes which must extend at least three rete ridges beyond the dermal component and (2) organization of this proliferation in a lentiginous or epithelioid-cell pattern. The minor criteria include: (1) presence of lamellar fibrosis or concentric eosinophilic fibrosis, (2) neovascularization, (3) an inflammatory response, and (4) the fusion of the rete ridges. Using these criteria, a diagnosis of dysplastic nevus requires both major criteria and at least two minor criteria.

The lesions were further analyzed for upwards spreading, mitoses and loss of maturation progression with depth combined with the presence of melanophages and inflammatory infiltrate. If a combination of these features was present these lesions were diagnosed as malignant melanoma [4, 10, 12, 17, 18, 21, 24]. Histopathologic descriptors are summarized in Table 1.

**Table 1:** Histopathology of melanocytic lesions compared with cross-sectional high-definition optical coherence tomography images

Subgroups	Common melanocytic nevi #13						
	JN	CNs	CNd	DeN	BN	DyN	MM
Descriptors	#3	#2	#6	#1	#1	#9	#4
Architectural							
General epidermal pattern							
Symmetry (Y/N)	Multiple images have to be taken as field of view is only 1.8 × 1.5 mm						
Circumscription (Y/N)							
Epidermis							
Acanthosis	3	2	6	0	0	3	3
Thining							1
Hyperpigmentation basal cell layer	2	1	5	1	0	8	2
Dermo-epidermal junction							
Rete Ridges							
Elongated	3	2	4	1	0	2	
Irregular	0	2	3	0	0	8	4
Fusion						7	3
Basilar proliferation extended three rete ridges beyond dermal component	0	0	0	0	0	9	
Lentigenous pattern						4	
Epitheloid-cell pattern						5	
Aggregates							
Junctional nests							
Regular, cohesive, uniform	3	2	6	0	0	4	
Irregular (with bridging and confluence)						5	4
Dermal nests							
Discrete nests expanding in papillary dermis	0	2					
Cohesive nests orderly aggregate in papillary/reticular dermis			6			1	
Orderly arranged nevus cells in the dermis arranged in nests or cords				1			
Discohesive nests						7	3
Ill-defined deep dermal proliferation of elongated/dendritic melanocytes					1		
Milia-like cysts			1	1			
Stroma reaction							
Plump bright cells (single or clusters)	2	2	5	1	1	9	4
Small bright cells	1	2	3	0	1	9	4
Neovascularization						7*	3
Fibrosis						7	3
Cytological atypia							
Atypical cells in nests, cords or solitary infiltrating the dermis (Y/N)	0	0	0	0	0	Y*	Y*
Presence of Pagetoid cells	0	0	0	0	0	0	4
Junctional nests with atypical cells						5*	4*
Dermal nests with atypical cells						2*	3*

*JN* junctional nevus, *CN* compound nevus, (s)uperficial and (d)eep type, *DeN* dermal nevus, *DyN* dysplastic nevus, *MM* malignant melanoma

^#^Number of patients included in each group

* Only observed if cross-sectional and en face imaging are combined

### Non-invasive imaging

Although the pigmented skin lesions were examined by an experienced dermatologist using only clinical, dermoscopic, and confocal information and without use of the histopathological diagnosis, a comparative review of the dermoscopic, confocal and HD-OCT images was performed with the histopathological information.

#### Dermoscopic description

The global dermoscopic pattern description was based on the previously described pattern analysis method [2, 3] and on the recently published classification of melanocytic nevi based on the integration of dermoscopic morphology and histopathology [18, 31]. The common nevi were classified according following dermoscopic patterns: reticular pattern, reticular-homogeneous pattern, reticular-homogeneous and/or globular pattern, complex-multicomponent pattern, cobblestone pattern, structureless blue pattern, and starburst pattern. The presence of atypical network, atypical peripheral globules of different size, peripheral streaks, structureless area with few branched streaks, blue-white veil overlying dark blotches, and atypical vascular patterns was registrated. According the ABCD rule, the Total Dermoscopy Score was estimated for each lesion. The seven-point checklist algorithm was also used for differentiating benign melanocytic lesions from melanoma based on pattern analysis [1].

#### Reflectance confocal microscopic pattern analysis of melanocytic lesions [14, 18, 23]

In the superficial epidermal layers, the general epidermal pattern (honeycombed, cobblestone or disarranged) was studied. At the dermo-epidermal junction, the overall architecture (ringed, meshwork with junctional thickening, clod pattern, or non-specific pattern) and the papillary contours (edged, non-edged, both edged and non-edged or totally disarranged) were evaluated. The presence of junctional nests (homogeneous dense/compact without atypical cells or inhomogeneous loose with atypical cells) and/or dermal nests (dens compact nests with monomorphic cells or dense and sparse nests with roundish monomorphic or loose aggregates of pleiomorphic cells) were registrated. Atypical roundish or dendritic cells were looked for. Pagetoide melanocytosis, characterized by the presence of atypical melanocytes in the upper part of the epidermis was assessed. The stroma reaction with plump bright cells solitary or in clusters corresponding with melanophages or small bright cells representing a lymphocytic infiltrate was evaluated together with blood-vessels and collagen structures. Table 2 summarizes RCM feature descriptors for melanocytic lesions.

**Table 2:** Reflectance confocal microscopy feature descriptors implemented on en face high-definition optical coherence tomography images

Subgroups	Common melanocytic nevi #13						
	JN	CNs	CNd	DeN	BN	DyN	MM
Descriptors	#3	#2	#6	#1	#1	#9	#4
Superficial epidermal layers							
General epidermal pattern							
Honeycombed pattern	1	1	1		1	1	
Cobblestone pattern	2	1	5	1		7	2
Disarranged pattern						1	4
Presence of Pagetoid cells	0	0	0	0	0	0	4
Hyperpigmentation basal cell layer	2	1	5	1	0	8	2
Dermo-epidermal junction							
Dominant overall architecture							
Ringed pattern							
Meshwork pattern	3	2	5	0	0	3	2
Clod pattern			3 (3)	1	0	6	
Non-specific pattern					1	5	2
Junctional thickening							
Elongated	3	2	4	1		2	
Irregular		2	3			8	4
Papillary contour					0		
Edged papillae				1			
Non-edged papillae	3	2	6			8	2
Totally disarranged						1	2
Aggregates							
Junctional nests							
Homogeneous dens/compact (no atypical cells)	3	2	6	0	0	4	
Inhomogeneous loose (with atypical cells)						5	4
Dermal nests							
Dense compact nests with monomorphic cells	0	0	6	1	0	1	
Dense and sparse nests							
With roundish monomorphic cells						5	
With loose aggregates of pleimorphic cells						2	3
Ill-defined deep dermal proliferation of elongated/dendritic melanocytes					1		
Milia-like cysts			1	1			
Atypical cells in nests, cords or solitary infiltrating the dermis	0	0	0	0	0	4 (few)	3
Stroma reaction							
Plump bright cells (single or clusters)	2	2	5	1	1	9	4
Small bright cells	1	2	3	0	1	9	4
Presence of fibrosis						7	3

*JN* junctional nevus, *CN* compound nevus, (s)uperficial and (d)eep type, *DeN* dermal nevus, *DyN* dysplastic nevus, *MM* malignant melanoma

^#^Number of patients included in each group

#### High-definition optical coherence tomography

The system is capable of capturing a cross-sectional image and an en face image in real time, as well as of fast 3D acquisition. The cross-sectional HD-OCT images were compared with histopathology as a validated reference. General histopathological descriptors of melanocytic lesions were compared with cross-sectional HD-OCT features (Table 1). A perfect correlation of structures is, however, not possible since the lesion were not studied with a punch biopsy of the exact area due to the guidelines regarding the diagnosis and treatment of pigmented lesions.

**Fig. 1:**
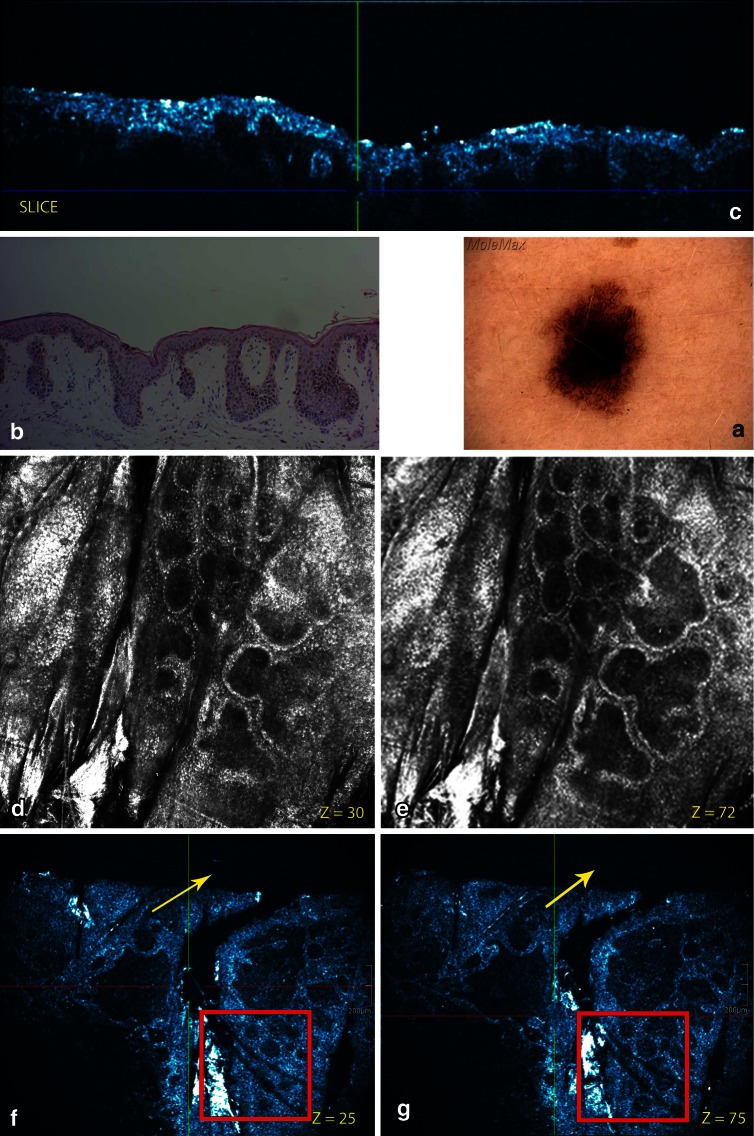
Common acquired melanocyt nevus, junctional nevus located on the back. **a** On dermoscopy, a wide irregular (atypical) pigment network (reticular pattern) can be observed. **b** Routine histopathology shows disarrangement of the rete ridges. Elongated and parallel oriented epidermal cristae are observed at the periphery of the lesion. Pigmented keratinocytes and transepidermal melanin loss is noticed. **c** The cross-sectional (cross-sectional) HD-OCT imaging demonstrates the pigmented rete ridges and bridging nests of melanocytic nests within the epidermis and papillary dermis as displayed in routine histopathology. **d**–**e** On the basic RCM images (*Z* = 30 μm *left* and *Z* = 72 μm *right*), a meshwork pattern is displayed with non-edged papillae. The dark dermal papillae are irregular in size and shape. No atypical cells are observed in these irregular junctional thickenings. Some compact, monomorphic dense nests are present. **f**–**g** On the corresponding en face HD-OCT images (*Z* = 25 μm and *Z* = 75 μm), the same features are seen despite the lower resolution. *Z* value = distance in μm from surface to focus location. *Red square* in HD-OCT image = corresponding field of view of RCM. On the HD-OCT en face images the border of a 2-mm diameter open plastic ring can be observed (*yellow arrow*)

Because melanocytic images were already validated on RCM, RCM feature descriptors for melanocytic lesions were implemented on en face HD-OCT images of different types of histological proven melanocytic lesions. To have an good correspondence between RCM and HD-OCT a 2-mm-diameter opening of a very thin plastic ring was positioned onto the skin directly after the dermoscopic images were obtained (Figs. 1, 5). By imaging melanocytic lesions with the HD-OCT a qualitative difference from normal skin was expected because the melanin present in melanocytes and nevus cells will result in higher reflectivity of these cells.

In vivo location and 3D-micro-architectural context of melanocytes and melanophages, solitary or in nests was defined by combining the cross-sectional and en face mode of HD-OCT and by using different color table settings (Figs. 6, 7). The characteristics of an individual cell or structure in the en face image can be checked on site by clicking with the cursor on this cell or structure in the en face image. The corresponding cross-sectional image of this cell or structure appears instantly. In combination with invert color table settings the architectural patterns and cytologic features of melanocytic lesions can be evaluated. These tools help in differentiating bright melanocytes from keratinocytes. These tools are also helpful in evaluating melanocytic maturation.

## Results

After histopathologic analysis by two board-certified histopathologists, the lesions of the 26 patients were classified as follows: 3 junctional nevi, 8 compound nevi (2 superficial type and 6 deep type), 1 dermal nevus and 1 blue nevus, 9 dysplastic nevi and 4 melanoma (superficial spreading melanoma all with Clark level II: 2 solar melanoma (Breslow 0.41 and 0.49 mm) and 2 pagetoid melanoma (Breslow 0.34 and 0.31 mm).

The features and definition of the structures seen in dermoscopy, RCM and histopathology are those described by Rajadhyasksha et al. and Scope et al. [25–27].

Histopathological and reflectance confocal microscopic feature descriptors of the melanocytic lesion types are displayed in Tables 1, 2.

### Dermoscopy and estimated total dermoscopy score (TDS)

**Fig. 2:**
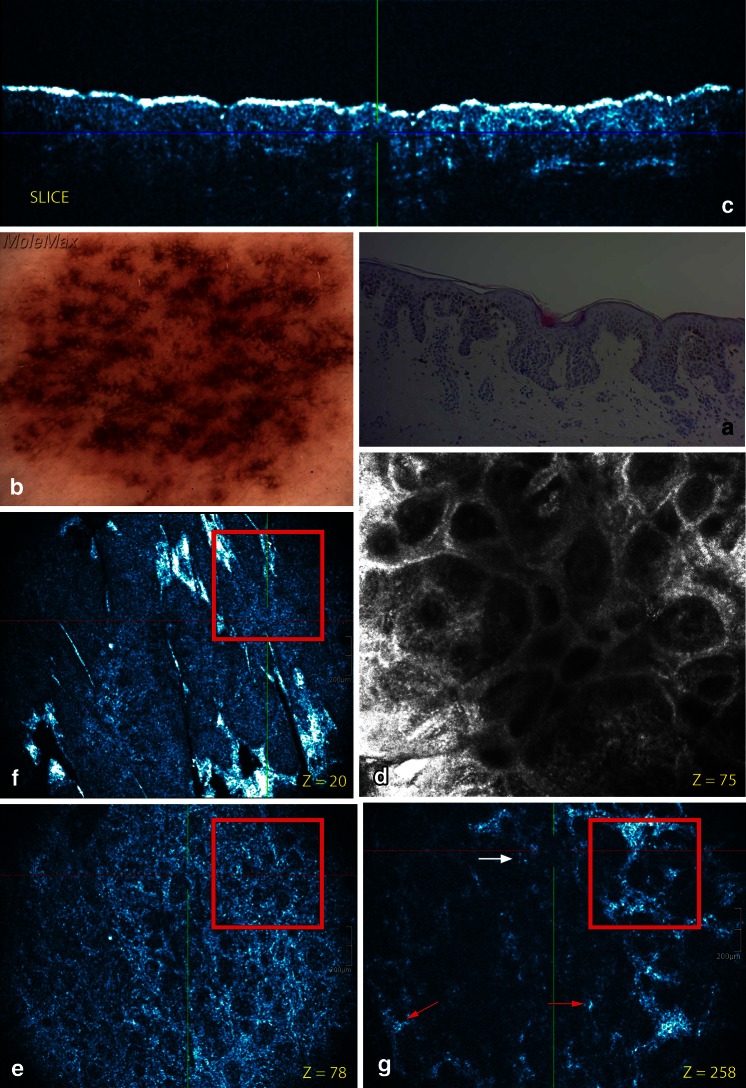
Common acquired melanocytic nevus, compound superficial type located on the back. **a** On dermoscopy, a typical pigment network (reticular pattern) and dark diffuse pigmentations (homogeneous pattern) are observed. **b** Routine histopathology shows elongated slightly irregular rete ridges with an increased number of melanocytes in the basal layers and hyperpigmented basal keratinocytes. Discrete melanocytic nests, composed by typical monomorphous cells located at the dermo-epidermal junction and within papillary dermis are noticed. Dermal nests and cords of monomorphic melanocytes are present. **c** The cross-sectional HD-OCT imaging permits the visualization of the increased number of melanocytes in the basal layers and hyperpigmented basal keratinocytes. **d** On the basic RCM image (0.5 × 0.5 mm) (*Z* = 75 μm), a mixed ringed and meshwork pattern can be observed. Roundish monomorphic cells are infiltrating the junctional layer. **e** On the corresponding en face HD-OCT image, the same features are present. **f** Higher in the epidermis a cobblestone pattern is noticed (*Z* = 20 μm). **g** Junctional nest and dermal nests with roundish monomorphic cells are observed even at a depth with *Z* value of 258 μm. Melanophages (*red arrow*) and inflammatory cells (*white arrow*) are observed

**Fig. 3:**
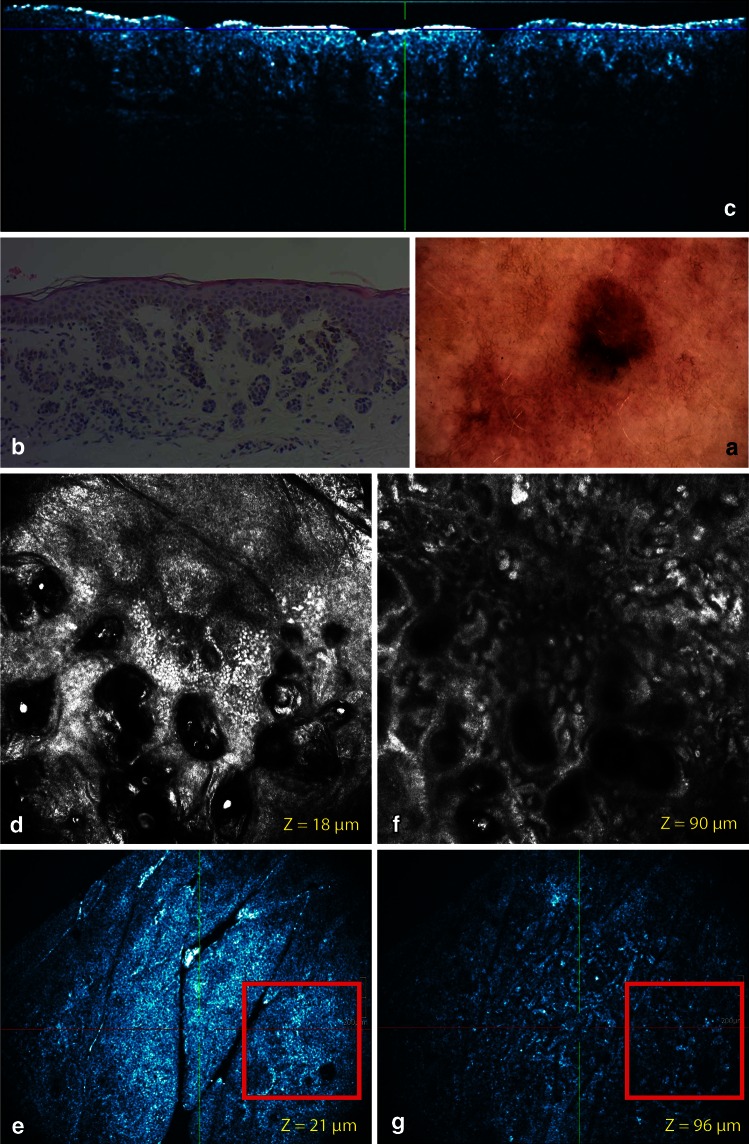
Common acquired melanocytic nevus, compound deep type located on the back. **a** On dermoscopy, a papillomatous melanocytic nevus with structureless areas is observed in close association to a seborrheic keratosis (SK). **b** On routine histology of the nevus elongated rete ridges are displayed. The distal tip of these ridges are often extended by junctional nests. Small junctional nests are also observed in the basal epidermis. A dermal proliferation of nevocytes in cords or nests is noticed. Epidermal inclusioncysts can be observed. **c** The corresponding cross-sectional HD-OCT imaging shows the elongated rete ridges which are very often extended at the distal tips. Small dermal nests are observed. **d** On the *left* basic RCM image (0.5 × 0.5 mm) taken at the border of the nevus and the SK, a cobblestone pattern of the epidermis is observed corresponding with the dark diffuse pigmentation of the nevus. Inclusion cysts are displayed. Follicular openings of the associated SK are observed. **e** On the corresponding en face HD-OCT image, the same features are present. **f** On the *right* basic RCM image (0.5 × 0.5 mm), a clod pattern is dominant. **g** On the corresponding en face HD-OCT image, a distinct clod pattern is displayed

**Fig. 4:**
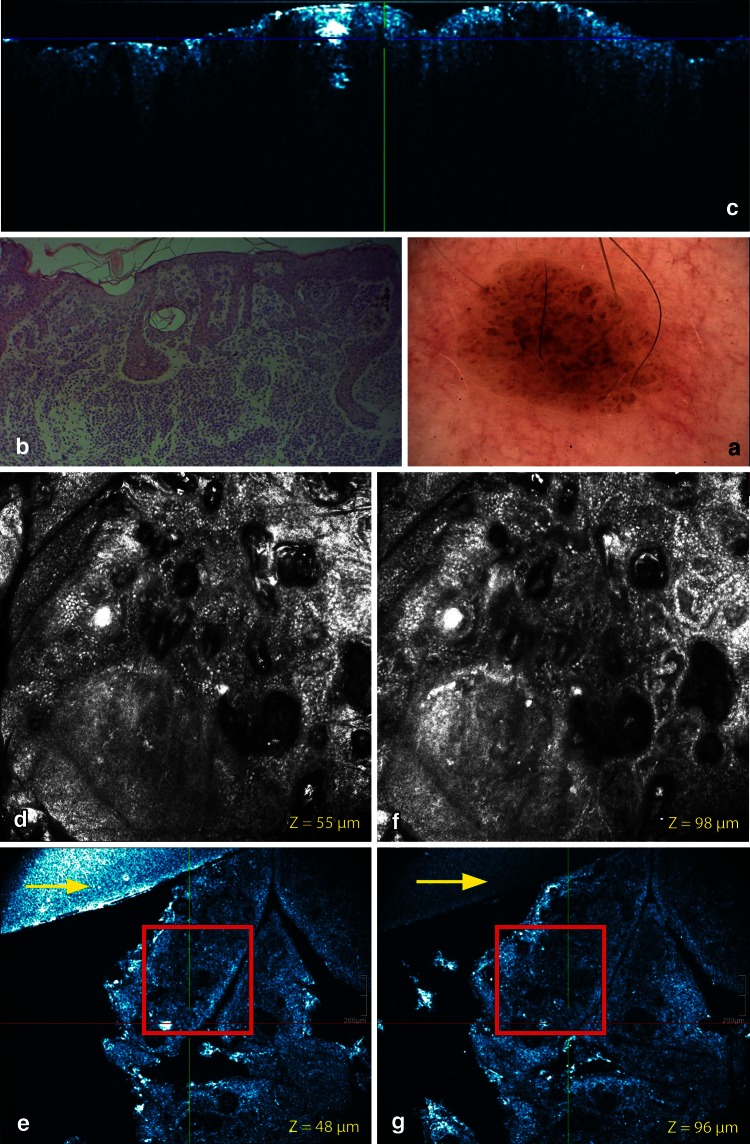
Common acquired melanocytic nevus, dermal nevus on the shoulder. **a** On dermoscopy, a cobblestone pattern can be observed. On routine histology elongated rete ridges are displayed. A dermal proliferation of nevocytes in cords or nests is noticed. Epidermal inclusioncysts can be observed. **c** The cross-sectional HD-OCT imaging shows the elongated rete ridges which are very often extended at the distal tips. Small dermal nests are observed. Inclusion cysts are also displayed. **d** On the *left* basic RCM image (0.5 × 0.5 mm) (*Z* = 55 μm), a cobblestone pattern of the epidermis is observed. Multiple inclusion cysts are displayed. **e** On the corresponding en face HD-OCT image, the same features are present. **f** On the *right* basic RCM image (0.5 × 0.5 mm) (*Z* = 98 μm), a clod pattern is dominant. **g** On the corresponding en face HD-OCT image, a distinct clod pattern is displayed. On both HD-OCT en face images, the border of a 2-mm diameter plastic ring can be observed (*yellow arrow*)

**Fig. 5:**
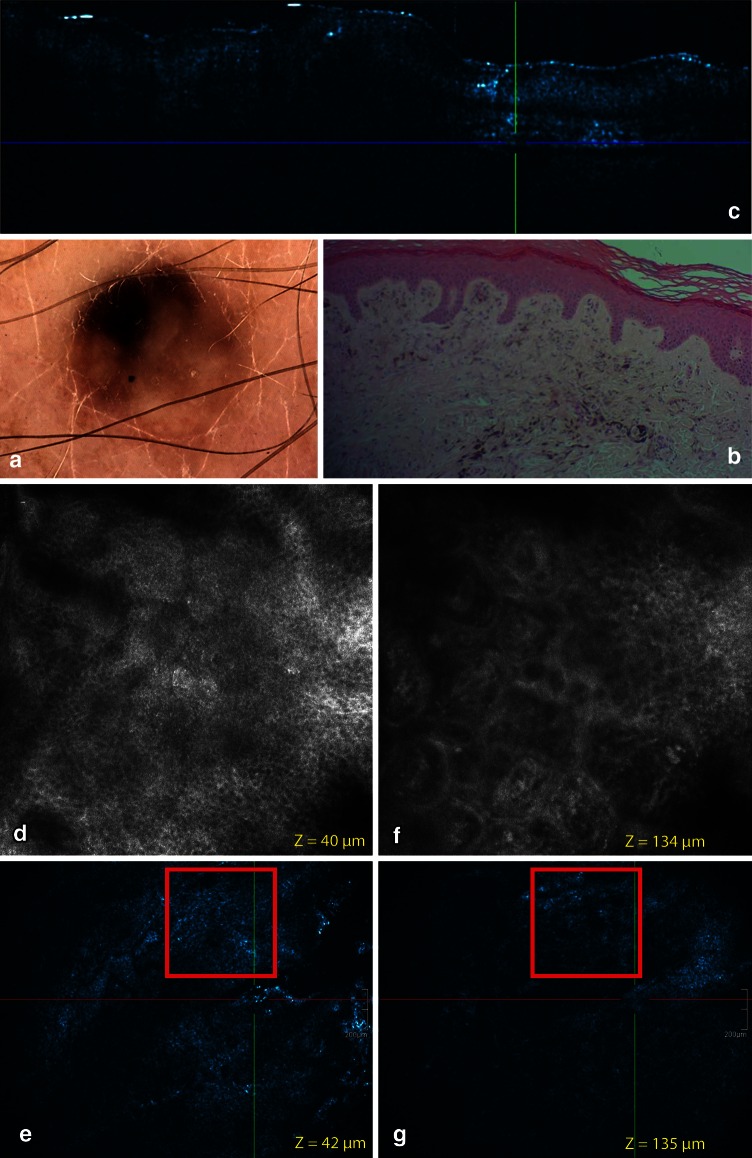
Common blue nevus on the dorsum of the *right* foot. **a** On dermoscopy, no typical *blue color* could be observed but rather a polychromatic structureless pigmentation. **b** On routine histology, a *thick* horny layer is covering a moderate acanthotic epidermis. In the dermis, a proliferation of markedly pigmented bipolar dentritic melanocytes dispersed as solitary units between thickened collagen bundles in the reticular dermis. Scattered pigment-laden melanophages are usually noted. **c** On the cross-sectional HD-OCT image, a thick stratum corneum is noted corresponding with the anatomic localization. The dermo-epidermal junction is preserved. Scattered bright cells deeper in the dermis are noted. **d** With confocal microscopy, a typical honeycomb pattern of the epidermis is preserved as demonstrated on the *left* basic RCM image (0.5 × 0.5 mm). **e** On the corresponding en face HD-OCT image, this typical honeycomb patter is also noted. **f** On the *right* basic RCM image (0.5 × 0.5 mm), scattered bright dendritic cells within the dermis are observed. These cells correspond to pigmented dendritic melanocytes. The main limitation of RCM in the assessment of common blue nevi is the restricted depth of penetration. **g** On the corresponding en face HD-OCT image, the scattered bright dendritic cells are displayed

**Fig. 6:**
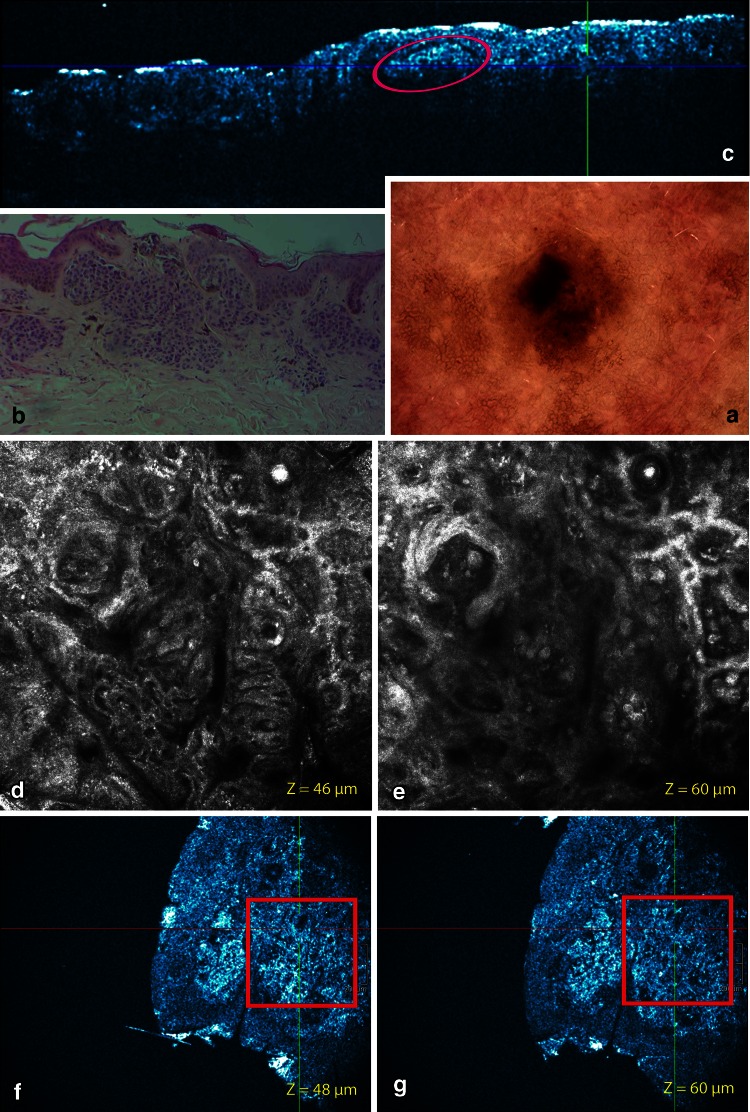
Severely dysplastic nevus, compound type located on the shoulder. **a** This eccentric hyperpigmented (blotches) nevus has a TDS of 6,1. **b** Routine histopathology shows dishomogeneous junctional nests with atypical cell infiltrating. A dermal proliferation of melanocytic cells in nests and cords. Progression maturation with depth is conserved. Mitoses are absent. No upwards spreading is observed. Hyaline fibroplasia is observed. Aggregates of melanophages and other inflammatory cells are displayed. **c** On the corresponding cross-sectional HD-OCT image, dishomogeneous junctional nests forming short interconnections and large dens nests in the upper dermis can be observed. Aggregates of melanophages are also present. **d**–**e** On the basic RCM images (0.5 × 0.5 mm) (*Z*-values 46 and 60 μm) in the center of the blotch dishomogeneous junctional nests with atypical melanocytes are displayed. A mixed appearance with non-specific pattern and clod pattern is observed. Roundish atypical cells are infiltrating the junctional layer. **f**–**g** Despite the lower lateral resolution, these features are also displayed on the corresponding en face HD-OCT image. Large dense and sparse nests with large roundish monomorphic cells are present in the upper dermis displayed on the en face HD-OCT image (1.8 × 1.5 mm) with *Z* value = 60 μm. Aggregates of plumb bright cells (melanophages) and small bright cells (lymphocytes) are noticed

**Fig. 7:**
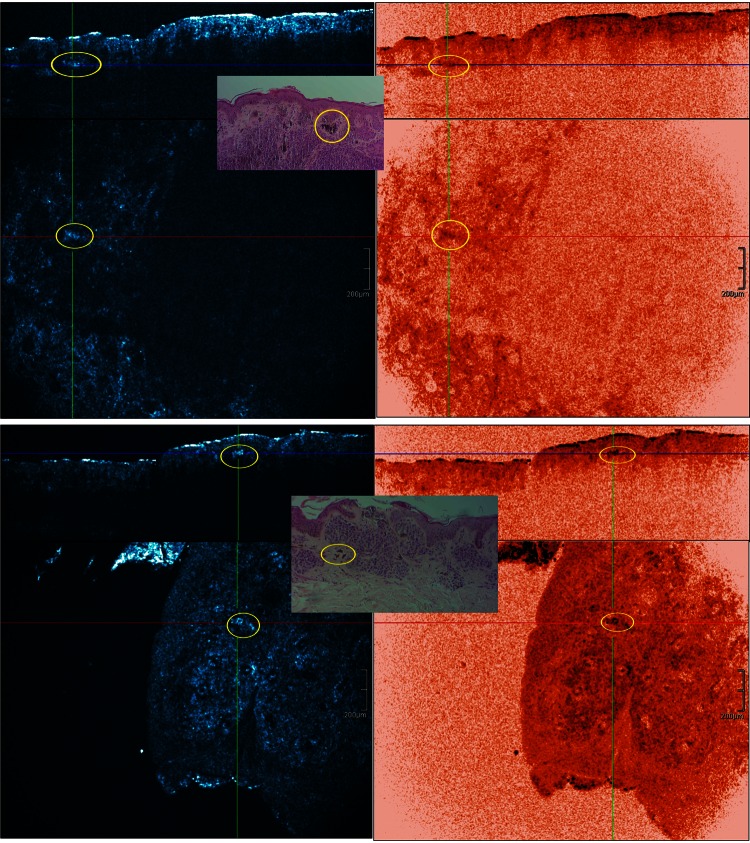
Same case as in Fig. 6. Imaging of nested growth pattern and cellular features of melanocytes and melanophages with different color table settings are possible. The combined use of normal (*left*) and invert color (*right*) palettes permits the identification and localization of melanin-rich cells and structures. Melanophages appear as large *oval* or stellate bright plump cells. The cytoplasmic border is mostly ill-defined. They are usually located as solitary cells or as loose aggregates of cells in the dermal papillae (*yellow encircled*)

**Fig. 8:**
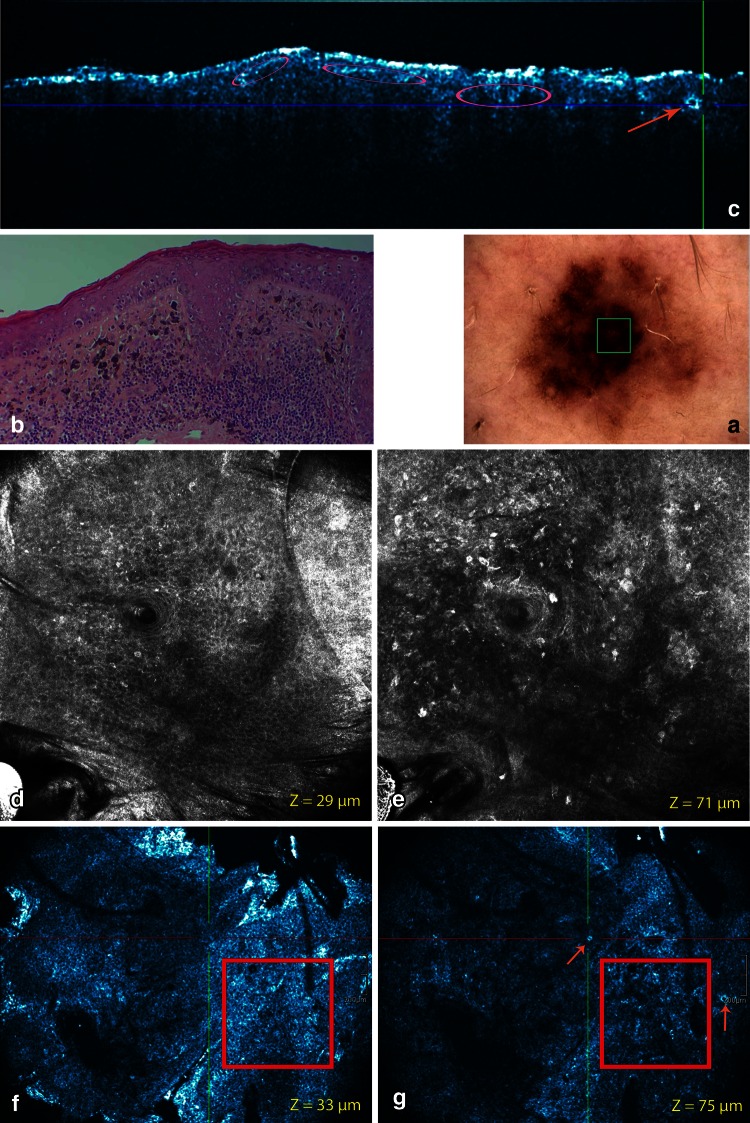
Solar melanoma neck. **a** This *brown* macule with eccentric atypical pigment network and a central perifollicular hyperpigmentation has a TDS of 6.8. **b** Routine histopathology shows proliferation of atypical melanocytic cells at the dermo-epidermal junction. These atypical cells tend to form junctional sheets. These cells are also present in the epithelium surrounding hairfollicles. A dermal proliferation of atypical melanocytic cells is observed. Progression maturation with depth is lost. Mitoses are observed. Focally upwards spreading is observed. Hyaline fibroplasia is present. Aggregates of melanophages and other inflammatory cells are displayed. **c** On the corresponding cross-sectional HD-OCT image dishomogeneous junctional nests can be observed (*pink encircled*). Melanophages are also present in the papillary dermis (*light brown arrow*). **d**–**e** On the basic RCM images (0.5 × 0.5 mm) (*Z*-values 29 and 71 μm) in the center of the blotch dishomogeneous junctional nests with atypical melanocytes are displayed. A non-specific pattern of the dermo-epidermal junction is observed. Roundish atypical cells are infiltrating the follicular epithelium. **f**–**g** Despite lower lateral resolution these features are also displayed on the corresponding en face HD-OCT image (*Z* values 33 and 75 μm)

A reticular/homogeneous pattern was present in three cases correlating well with the histopathological junctional nevus type (Fig. 1). A reticular/homogeneous/globular pattern was observed in two cases corresponding with the histopathological compound nevus superficial type (Fig. 2). A complex/multicomponent pattern was present in the histopathological compound nevus deep type (6 cases) (Fig. 3), A cobblestone pattern was present in the dermal nevus (1 case) (Fig. 4) and a structureless blue pattern was observed in the blue nevus (1 case) (Fig. 5). A complex/multicomponent pattern was observed in the dysplastic nevi (9 cases) (Figs. 6, 7) and in the four melanoma cases (Fig. 8).

The combination of atypical network, atypical peripheral globules of different size, peripheral streaks or structureless area with few branched streaks, blue-white veil overlying dark blotches and atypical vascular pattern was only observed in melanoma. The mean TDS value of common acquired melanocytic lesions, dysplastic nevi and superficial spreading melanoma was calculated and was 4.66, 5.72 and 6.53, respectively. According the 7-point checklist based on pattern analysis, only the malignant melanoma lesions scored higher than 3.

### HD-OCT descriptors for melanocytic lesions

#### Common melanocytic nevi

By imaging these melanocytic lesions with the cross-sectional and en face mode of HD-OCT a qualitative difference from normal skin could be observed.

##### Cross-sectional mode

In this mode an altered overall architecture of the dermo-epidermal junction was observed with elongated and broadened rete ridges. Rete ridges were elongated in 11/13 and irregular in 5/13 cases. Elongated and irregular rete ridges were both present in the same patient in 3/13 cases. Fusion of rete ridges was occasionally observed. Junctional and/or dermal cellular aggregates could be observed. These findings corresponded with routine histopathology (Table 1).

##### En face mode

The cobblestone pattern was the predominant epidermal pattern in melanocytic lesions (9/13) (Figs. 1, 2, 3). In the absence of this hyperpigmentation a honeycomb pattern was observed (4/13). No pagetoid cells were observed. The most striking observation in common acquired melanocytic nevi was the presence of small, bright, highly reflective cells in the dermo-epidermal junction and/or dermis in the HD-OCT en face mode. Depending on the image focus these cells appeared in this mode either homogeneously bright with dark nuclei within or as bright disks without observable nuclei. In melanocytes, the signal intensity was generally present throughout the entire cytoplasm in a uniform pattern. At the dermo-epidermal junction, a meshwork pattern was most frequently noticed (11/13) (Fig. 2). A clod pattern was observed in the case of dermal nevus (Fig. 4) and a non-specific pattern was observed in the case of blue nevus (Fig. 5). Non-edged papillae were noticed in 11/13 cases (Fig. 2). Despite the difference in lateral resolution these en face HD-OCT findings corresponded with RCM findings (Table 2).

##### Combined cross-sectional and en face mode

In vivo location of melanocytes, solitary or in cluster, could be defined by combining the cross-sectional and en face mode and by using different color table settings (Tables 1, 2).

Nested growth patterns of melanocytes could be recognized. Some melanocytes were brighter than others. Distinct cellular outlines were not always visible within nests. The bright signals of individual cells frequently fused and formed a spherical structure of variable size and shape with no further cellular details. The cellular clusters could be focally aggregated or diffusely distribute depending on either their presence in limited areas of the lesion or their diffuse presence on the whole investigated area. The clusters could be small (major axis inferior to 250 μm) (Figs. 1, 2, 3), medium (from 250 to 500 μm) or large (over 500 μm). Cellular clusters were also different according to their aspect. Some clusters formed compact hyper-reflecting aggregates of polygonal cells with hyporeflective nuclei (Fig. 3). Others presented as roundish lower-reflecting structures with a well-circumscribed border. They contained isolated cells with a dark nucleus and reflecting cytoplasm (Fig. 1).

Homogeneous dense and compact junctional nests without atypical cells were observed in 11/13 cases. Maturation of the melanocytes could be evaluated. When melanocytes descended into the dermis they gradually diminished in size with less apparent nuclei. Moreover, in the papillary dermis nests were large and in the deeper dermis nests became smaller and eventually only single melanocytes were found. No junctional nests were observed in the dermal nevus. In this type of nevus dense compact nests with monomorphic cells were observed (Fig. 4). This was also the case for the deep type compound nevi (6/13) (Fig. 3). Milia-like cysts could be noticed in the dermal nevus and some deep compound nevi. In the case of the blue nevus a ill-defined deep dermal proliferation of elongated/dendritic melanocytes was observed (Fig. 5).

Melanophages presented as solitary cells or loose aggregates of cells in the dermal papillae under RCM. These large oval or stellate bright plump cells were observed by both HD-OCT en-face images and corresponding cross-sectional images. These cells were usually larger than individual melanocytes. These variably bright cells generally have fuzzy, ill-defined cell borders. They were usually located as solitary cells or as loose aggregates of cells in the dermal papillae around or near bloodvessels. These cells were observed in 11 out of the 13 common melanocytic nevi (Fig. 7). In six of these lesions small bright cells corresponding with inflammatory cells could be seen (Figs. 2, 7).

#### Dysplastic nevi

##### Cross-sectional mode

Disarranged rete ridges were observed. Fusion or interconnections of rete ridges were frequently noticed (7/9). In all cases except one both junctional and dermal nests were observed. Basilar proliferations extending beyond the dermal component were always present. These proliferations were organized in a lentiginous (4/9) or a epitheloid-cell pattern (5/9). Presence of a dark belt at the dermo-epidermal junction and upper papillary dermis was noticed in 7/9 case, probably correlating with concentric eosinophilic fibrosis observed on histopathology (Table 1).

##### En face mode

The cobblestone pattern was the prominent pattern (7/9). There was one case with a honeycomb pattern and one with a disarranged pattern. No pagetoid cells were observed. The dominant overall architecture of the dermo-epidermal junction was a clod pattern in 6/9 sometimes associated with a meshwork pattern in 3/9 cases or with a non-specific pattern in 5/9 cases (Figs. 6, 7). The junctional thickenings were irregular. The papillary contours were characterized by non-edged papillae in 8/9 cases. In one case totally disarranged papillary contours were observed (Table 2).

##### Combined cross-sectional and en face mode

The basilar proliferation of atypical melanocytes could be organized in a lentiginous or epitheloid-cell (Fig. 6) pattern. Homogeneous dense/compact junctional nests without atypical cells were observed in 4/9 cases. In one of these cases the dermal nests were compact with monomorphic cells. In 5/9 cases the junctional nests were inhomogeneous loose with atypical cells. Dense and sparse dermal nests with roundish monomorphic cells were observed in 5/9 cases. In 2/9 cases, the dense and sparse dermal nests were characterized by loose aggregates of pleomorphic cells. In one case no dermal nests were observed. Atypical cells in nests, cords or solitary infiltrating the dermis could be noticed in 4/9 cases. All the dysplastic nevi presented a stromal reaction with plump bright cells solitary or in clusters corresponding with melanophages and small bright cells representing a lymphocytic infiltrate (Figs. 6, 7). Neovascularization was often noticed. Imaging of a nested growth pattern of melanocytes with different color table settings is possible. The combined use of both palettes permits the identification and localization of melanin-rich cells and structures (Fig. 7; Tables 1, 2).

#### Superficial spreading melanoma

##### Cross-sectional mode

**Fig. 9:**
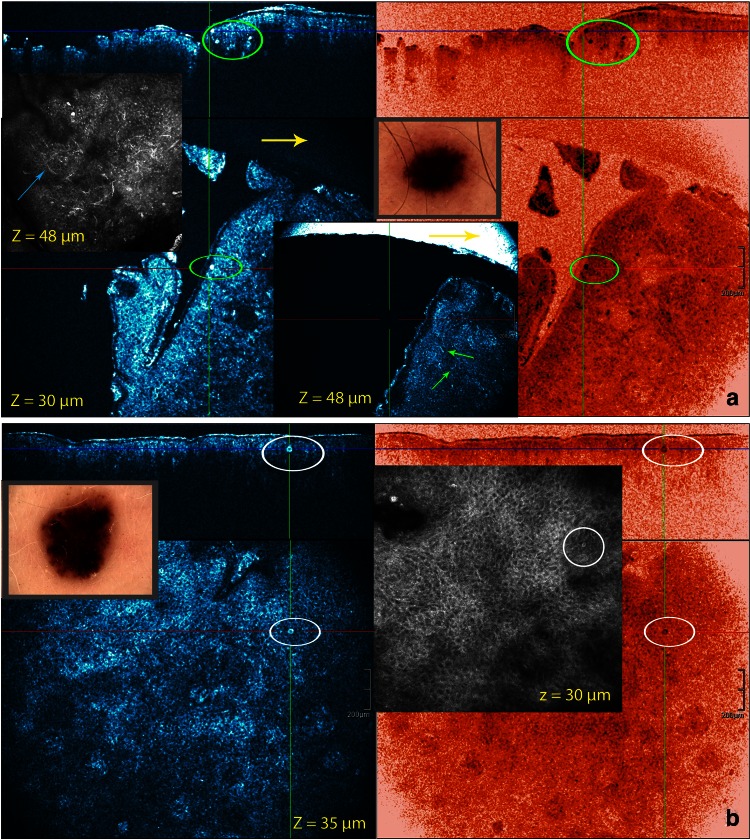
Two cases of pagetoid melanoma leg. **a**, **b** The polymorphous complex dermoscopic pattern of the lesion results in a high TDS. **a**, **b** Pagetoid melanocytosis is represented by the presence of large melanocytes (twice than a neighboring keratinocyte) with abundant reflective cytoplasm (*green encircled*) and sometimes characterized by a prominent hyporeflective nucleus depending on the image focus (*white encircled*). **a** The pagetoid cells may be more elongated with variable morphology of the branches. *Thick* branches can still be observed by HD-OCT (*green arrow*). *Thin* bright filamentous structures are only observed by RCM (*blue arrow*). **a** On the HD-OCT en face images, the 2-mm diameter plastic ring can be observed (*yellow arrow*)

Atypical melanocytes could be observed in the upper part of the acanthotic epidermis. These large melanocytes (twice than a neighboring keratinocyte) had an abundant reflective cytoplasm (Fig. 9 upper part). A non-specific pattern of the overall architecture of the dermo-epidermal junction was observed with irregular and broadened rete ridges. The atypical cells tended to form junctional sheets (Fig. 8) or irregular junctional aggregates distorting the rete ridges. In one case signs of actinic damage were present: thinning of the epidermis and solar elastosis (Table 1).

##### En face mode

The general pattern of the superficial layer could be cobblestone or irregular honeycomb pattern. The more pronounced the pigmentation the higher the likelihood of finding a cobblestone pattern. An epidermal disarray was observed in areas of pagetoid spread. Atypical melanocytes were observed in the upper part of the epidermis. These large melanocytes (twice than a neighboring keratinocyte) had an abundant reflective cytoplasm (Fig. 9 upper part) and were sometimes characterized by a prominent hyporeflective nucleus depending on the image focus (Fig. 9 lower part). In one case these pagetoid cells were more elongated with variable morphology of the branches. On corresponding RCM images thick and short or thin bright filamentous structures could be observed. On HD-OCT only the thick and short filamentous structures were discernable. The cell distribution was localized because only in a small portion of the lesion pagetoid cells could be detected. The use of different color table settings permits the identification and localization of pagetoid cells in the epidermis (Fig. 9; Table 2).

##### Combined cross-sectional and en face mode

In two cases, a non-specific pattern of the overall architecture of the dermo-epidermal junction was observed. In those cases, the atypical cells tended to form junctional sheets (Fig. 8). In the two others cases, a meshwork pattern determined the architecture of the dermo-epidermal junction. The junctional melanocytic aggregates distorted the rete-ridges giving rise to inhomogeneous loose junctional nests with atypical cells. Dermal dense and sparse nests with loose aggregates of pleiomorphic cells were observed in 3/4 cases (Figs. 8, 9).

A loss of maturation progression with depth could be observed by the combined use of the en face and cross-sectional HD-OCT images. The use of invert color table setting permits the user to double check these findings. Melanocytes deeper in the dermis were indistinguishable from those within the superficial papillary dermis. All the SSM presented a stromal reaction with plump bright cells solitary or in clusters corresponding with melanophages and small bright cells representing a lymphocytic infiltrate (Fig. 8; Tables 1, 2).

## Discussion

This study indicates that HD-OCT potentially allows the identification of the architectural patterns and cytologic features of pigmented cells in the epidermis, dermo-epidermal junction, and dermis up to the superficial reticular dermis. Although the HD-OCT images of pigmented skin lesions were examined by an experienced dermatologist using only clinical, dermoscopic, and confocal information and without use of the histopathological diagnosis, subsequent interpretation of the HD-OCT images was grounded with the histopathological information adding a standard point of reference.

A good correspondence of cross-sectional HD-OCT images of melanocytic nevi with histopathologic features could be observed (Table 1).

Despite the difference in lateral resolution, most of the RCM features of different types of melanocytic lesions could be observed on the en face HD-OCT images (Table 2). Therefore, RCM feature descriptors for melanocytic lesions were implemented on en face HD-OCT images (Table 2). We have previously explored the implementation of validated RCM features/terminology in HD-OCT and strongly believe that the use of uniform terminology is to the advantage of both methods [4–7].

By combining in real time, the cross-sectional and en face mode (3D), in vivo on site 3D-location of melanocytes, solitary or in cluster, and their differentiation from keratinocytes could be defined (Figs. 6, 7). Imaging of a nested growth pattern of melanocytes with different color table settings is possible. The combined use of both palettes permits the identification and localization of melanin-rich cells and structures (Fig. 7).

High-definition optical coherence tomography may therefore potentially develop into one of the adjuvant methods used in the diagnosis of malignant melanoma. Although an experienced dermatologist can accurately diagnose most melanocytic lesions on the basis of clinical and dermoscopic criteria alone, RCM has already proven to be a valuable adjunct to clinical and dermoscopic examination [19–27].

High-definition optical coherence tomography has important limitations. First, the lateral resolution is approximately 1/3 that of RCM. Hence, dendritic-like processes having a diameter of <3 μm cannot be observed by HD-OCT (Fig. 9). Secondly, making a mosaic over an 8 mm square area (= 256 times a field of view) as is possible with RCM VivaScope 1500^®^ is impossible with HD-OCT and handheld RCM VivaScope 3000^®^. The field of view of HD-OCT is 1.8 × 1.5 mm compared to the 0.5 × 0.5 mm field of view of handheld RCM making of the HD-OCT en face image a mini-mosaic of almost 11 RCM fields (0.5 × 0.5 mm) of view. Furthermore, RCM allows the observation of vascular flow and rolling of leukocytes in vessels which are not visible with HD-OCT static images. In special anatomic areas, mainly on the concave areas of the face and interdigital spaces, the probe of the HD-OCT cannot physically be placed on the skin surface due to the size of the probe. In contrast using handheld RCM allows examination of difficult anatomic sites, but not of structures located deeper than 250 μm.

Further studies need to be performed in an observer-blinded manner to objectively validate the HD-OCT findings and establish the clinical utility of the method. This pilot study gives valuable insight to strengths and limitations of HD-OCT which need to be addressed in the design of the full-scale validation studies.

In conclusion, this study suggests that HD-OCT may provide morphological imaging with sufficient resolution and penetration depth to discriminate architectural patterns and cytologic features of pigmented lesions and cells in the epidermis, papillary dermis and even superficial reticular dermis, adding relevant 3D-structural information complementary to that of other imaging methods.
